# Diabetes and obesity and risk of pyogenic liver abscess

**DOI:** 10.1038/s41598-023-34889-z

**Published:** 2023-05-16

**Authors:** Jiun-Ling Wang, Chun-Ru Hsu, Chieh-Yin Wu, Hsien-Ho Lin

**Affiliations:** 1 Department of Internal Medicine, National Cheng Kung University Hospital and College of Medicine, National Cheng Kung University, Tainan, Taiwan; 2 Department of Bioscience and Biotechnology, National Taiwan Ocean University, Keelung, Taiwan; 3 Institute of Epidemiology and Preventive Medicine, National Taiwan University, Taipei, Taiwan

**Keywords:** Risk factors, Gastrointestinal diseases, Infectious diseases, Metabolic disorders, Epidemiology

## Abstract

Few literatures discussed the relationship of glycemic control and body mass index (BMI) with the risk of pyogenic liver abscess. We conducted a population-based cohort study using participants of a community-based health screening program in Taiwan from 2005 to 2008 (n = 125,865). Information on fasting plasma glucose (FPG), BMI, and other potential risk factors of liver abscess were collected at baseline. Incidence of pyogenic liver abscess was ascertained using inpatient records from the National Health Insurance database. During a median 8.6 years of followed up, 192 incident cases of pyogenic liver abscess were reported. The incidence rate of pyogenic liver abscess was 70.2 and 14.7 per 100,000 in the diabetic and non-diabetic population respectively. In multivariable Cox regression analysis, the adjusted hazard ratio (HR) was 2.18 (95% confidence interval (CI) 1.22–3.90) in patients with diabetes with good glycemic control (FPG ≤ 130 mg/dl) and 3.34 (95% CI 2.37–4.72) in those with poor glycemic control (FPG > 130 mg/dl), when compared with non-diabetics. In the dose–response analysis, the risk of liver abscess increased monotonically with increasing FPG. After adjusting for diabetes and other comorbidities, overweight (25 ≤ BMI < 30) (adjusted HR: 1.43, 95% CI 1.05–1.95) and obese (BMI ≥ 30) (adjusted HR: 1.75, 95% CI 1.09–2.81) populations had a higher risk of liver abscess when compared to people with normal weight**.** Diabetes, especially poorly controlled disease, and high BMI were associated with higher risk of pyogenic liver abscess. Improving glycemic control and weight reduction may reduce the risk of developing pyogenic liver abscess.

## Introduction

Over the past few decades, the prevalence of diabetes has been increasing^1^. In previous large diabetic cohorts, infection was a major cause of mortality^2,3^. Few studies have discussed the relationship between glycemic control and infectious diseases in the diabetic population^4^. A number of studies reveal that poor glycemic control is correlated with post-operative infections^5,6^. However, there is a paucity of research discussing the relationship between glycemic control and the risk of community-acquired infection, including intra-abdominal infections as pyogenic liver abscess. Starting in the 1980s in Taiwan and Asia, a distinct syndrome resulting from *Klebsiella pneumoniae* infection and marked by pyogenic liver abscess began to appear in anecdotal literature^7^, although it has since emerged as condition of global concern^8–10^. In Taiwan, the incidence of pyogenic liver abscess increased steadily from 1996 to 2011^11^. Diabetes and malignancy were the most well-known risk factors for the disease^12^. Among the Taiwanese population, population-based pyrogenic liver abscess mortality is approximately 10%, while in-hospital mortality is 6%^12^. At present, tt is unknown if better glycemic control would lead to decreased incidence of pyogenic liver abscess among patients with diabetes.

Besides diabetes, the prevalence of obesity has also increased rapidly around the world^13^. Limited data suggests that obesity and high body mass index (BMI) increased infection risk in adults, but most of the studies focus on surgical site infections and other nosocomial infections^14^. Meanwhile, confounding factor such as medical co-morbidities may introduce to bias when considering the effect of obesity on infection risk. A recent mice animal model mice found obesity may impair host defense against *Klebsiella pneumoniae*^15^, which is the most important pathogen of pyogenic liver abscess. Similar to diabetes, there is a lack of literature regarding the association between BMI and the risk of pyogenic liver abscess.

Both diabetes and obesity have the potential to lead to immune system dysfunction e.g. hinder chemotaxis, modify macrophage differentiation^16,17^. And obesity increase the likelihood of developing comorbidities such as diabetes, which may, in turn, heighten the risk of bacterial infection. We hypothesized that poor glycemic control and high BMI may be associated with higher risk of pyogenic liver abscess. In this study, we aimed to investigate the association between diabetes mellitus, glycemic control and the risk of incident pyogenic liver abscess. We also examined whether people with a higher BMI had a higher risk of pyogenic liver abscess compared to their counterparts with a normal-range BMI.

## Methods

### Study design

**Figure 1:**
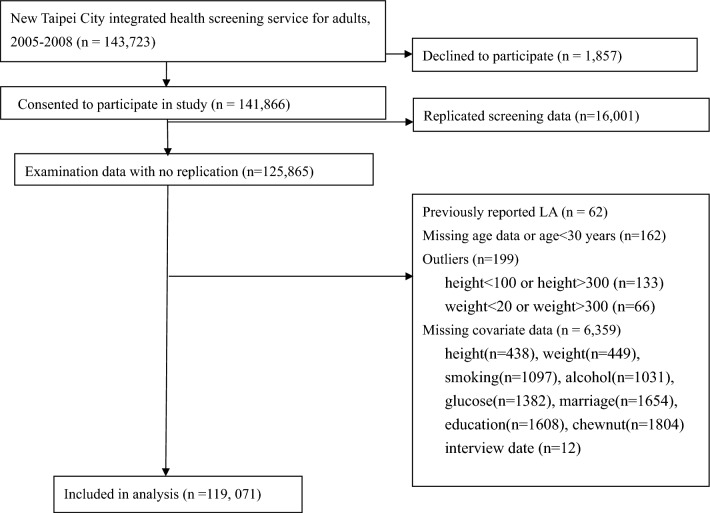
Flow diagram of inclusion and exclusion of study participants.

We assembled a cohort using participants from a community-based health screening service (n = 125,865). The service is a voluntary and free health screening program for the residents aged 30 years or older in New Taipei City from 2005 to 2008. The participants filled out a questionnaire on demographics and lifestyle information and received biochemical blood test. All individuals who were enrolled in the program and consented to participate were included in the study. Details of the cohort have been described in detail in previous studies^18,19^. The screening program database was cross-matched to the National Health Insurance database using the unique national identification number. In Taiwan National Health Insurance was compulsory for all residents, and the coverage rate is over 99%^20^. After excluding 6794 participants with previous pyogenic liver abscess or missing covariate information, 119,071 were included in the final analysis (Fig. 1). This study was approved by the Research Ethics Committee of the National Taiwan University Hospital, Feb 2016 (201601058W) and waived the need for informed consent. All research was performed in accordance with relevant guidelines/regulations.

### Measurement of diabetes and body mass index

Diabetes and BMI were the main exposures of interest in this study. Patients with diabetes were identified if any of the following criteria were met: (1) fasting plasma glucose (FPG) over 126 mg/dL; or (2) prescription of hypoglycemic agent (verified from the health insurance claims database) for more than 28 days in the past 2 years. Additionally, we used the Diabetes Complications Severity Index (DCSI) to measure disease severity in each diabetic patient. The DCSI was estimated based on the number of diabetes-related complications reported at baseline using the information from national health insurance data^21^. BMI was calculated by dividing weight (in kilograms) by height squared (in meters). Weight and height were self-reported by the participants. We categorized BMIs following the WHO classification into groups of underweight (< 18.5 kg/m^2^), normal (18.5–25.0 kg/m^2^), overweight (25.0–30.0 kg/m^2^), and obese (≥ 30.0 kg/m^2^)^22^.

### Measurement of pyogenic liver abscess

The primary outcome of interest was the incidence of pyogenic liver abscess. Occurrence of liver abscess was ascertained from the national health insurance claims database. We defined liver abscess based on hospitalization record with a compatible discharge diagnosis. The cohort participants were followed until diagnosis of pyogenic liver abscess, death (based on vital registry), or the end of 2014, whichever came first. The discharge diagnoses included abscess of liver per International Classification of Diseases, 9th revision, Clinical Modication (ICD-9-CM 572.0) but excluded amebic liver abscess (ICD-9-CM 006.3). In Taiwan, the diagnosis of pyogenic liver abscess is usually based on clinical symptoms, imaging such as abdominal sonography and/or CT scan and bacterial culture from blood or pus aspiration . In a previous study of patients diagnosed with pyogenic liver abscess in Taiwan, *Klebsiella pneumoniae* was isolated in about 80% of cases^12^.

### Measurement of other covariates

In our analysis we accounted for known risk factors of pyogenic liver abscess which we identified through literature review. Baseline demographics information (e.g. sex, age, level of education, and marriage status) and behavioral risk factors (e.g. smoking and alcohol use) were obtained from the questionnaire completed at cohort entry. Information on relevant medical comorbidities (cirrhosis, biliary tract and gallbladder disease, and gastrointestinal cancer) was obtained from the national health insurance database. Causal diagrams were used to determine which variables should be adjusted for in the multivariable analysis(See Appendix Figure 1)^23^.We did not adjust for or exclude chronic kidney disease since we thought chronic kidney disease is an intermediate variable in the causal pathway between diabetes and pyogenic liver abscess.

### Statistical analysis

We tabulated the frequency distribution of baseline characteristics in the full cohort as well as by diabetes status (no diabetes, diabetes with good control, and diabetes with poor control). Diabetes with good control was defined as FPG ≤ 130 mg/dL, while diabetes with poor control was defined as FPG > 130 mg/dL^24^. Kaplan–Meier curves were plotted to assess time to onset of liver abscess, and the log rank test was used to test for difference in liver-abscess-free survival among the different diabetes groups. We used Cox regression models to estimate the hazard ratios (HRs) and 95% confidence intervals for different levels of diabetes (by FPG or DCSI) control and BMI, adjusting for other covariates.

To explore the potential of a non-linear relationship between glycemic control (FPG) and risk of liver abscess as well as that between BMI and risk of liver abscess, we estimated the non-linear associations using penalized spline regression with three degrees of freedom^25^. Tests of non-linearity were conducted using likelihood ratio tests comparing the non-linear model to the linear model. We also performed subgroup analysis to investigate effect modification of diabetes by age, sex, BMI, and biliary disease. Cross-product terms were created and added to the multivariable Cox model and models with and without cross-product interaction terms were compared using the likelihood ratio test. We estimated the proportion of cases of liver abscess that could be attributed to diabetes and high BMI (overweight/obesity) by calculating the population attributable fraction using the Levin’s formula^26^. We tested the proportional hazards assumption for Cox regression model using the *cox.zph()* function in the survival package of R. All analyses were conducted using SAS software version 9.4 (SAS Institute, Cary, North Carolina) and R software version 3.1.2 (R project).

### Ethics approval

This study was approved by the Research Ethics Committee of the National Taiwan University Hospital, Feb 2016 (201601058W) and all research was performed in accordance with relevant guidelines/regulations.

## Results

The study population had a median age of 51 years and was predominantly female (64.4%). Of the total 119,071 study participants, 10,745 (9.0%) had diabetes at baseline, and 7771 of the diabetics had poor glycemic control (72.3% of the diabetic population). Compared to those without diabetes, the diabetic populations were more likely to be elderly, have higher BMI, be alcohol users, and have lower educational attainment.

During a median follow up of 8.6 years, 192 incident cases of liver abscess were reported. Of these patients with liver abscess, four had metastatic complications and one died with the underlying cause of death coded as liver abscess. The overall incidence rate of liver abscess was 19.6 per 100,000 person-years (95% CI 16.8–22.4 per 100,000). The incidence rate of liver abscess was substantially higher in the diabetic population (70.2 per 100,000) than in the non-diabetic population (14.7 per 100,000). Within the diabetic population, the incidence rate was higher in those with poor glycemic control (75.7 per 100,000) than those with good glycemic control (55.7 per 100,000). With regard to BMI status, the incidence rate of liver abscess was lowest in the normal weight and underweight groups (14.2 and 11.0 per 100,000 respectively). The incidence rate increased with increasing BMI: 26.9 per 100,000 in the overweight population and 34.6 per 100,000 in the obese population.

**Figure 2:**
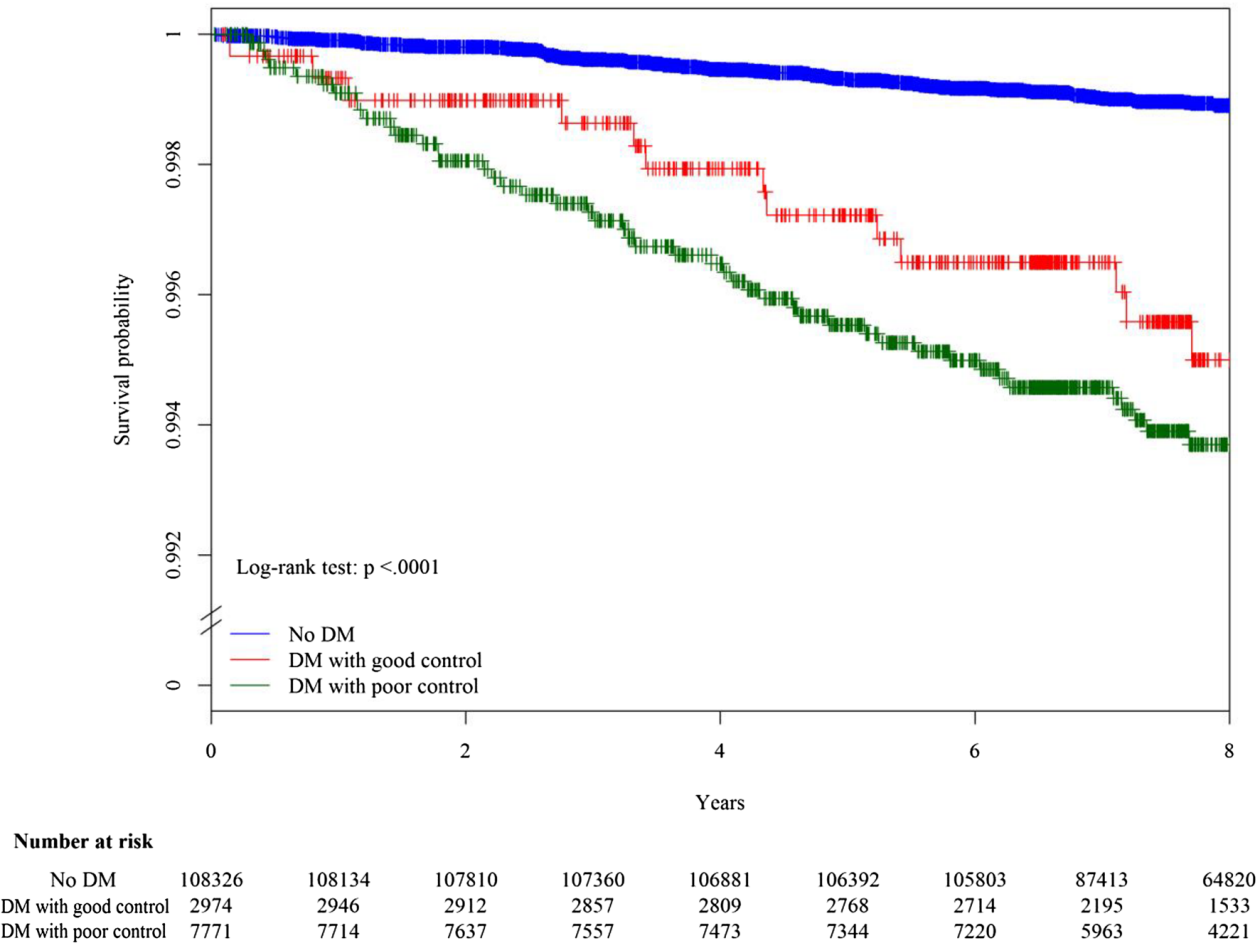
Kaplan–Meier plot of pyogenic liver abscess-free survival in the absence of death by different status of diabetes mellitus (DM) and glycemic control. Good glycemic control: fasting plasma glucose ≦130 mg/dL. Poor glycemic control: fasting plasma glucose > 130 mg /dL.

In the Kaplan–Meier plot, the liver-abscess-free survival was different among those with different diabetes status (log-rank test: p < 0.001) (Fig. 2). The liver-abscess-free survival was worst within the diabetic population with poor glycemic control. In the age- and sex-adjusted Cox regression analysis, diabetes was associated with an increased risk of liver abscess, and the risk increased among diabetic people with poor glycemic control compared to diabetic people with good glycemic control (Table 1). In the multivariable-adjusted Cox regression analysis, the relation between glycemic control and risk of liver abscess remained unchanged. Compared with non-diabetics, the adjusted HR was 2.18 (95% CI 1.22–3.90) in diabetics with good glycemic control and 3.34 (95% CI 2.37–4.72) in those with poor glycemic control. When diabetic patients were classified based on the severity of diabetes (DCSI), the risk of liver abscess did not vary substantially among diabetic patients with different DCSI (Table 1). In the subgroup analysis, there was evidence of effect modification by age (p value: 0.017). The association between glycemic control and risk of liver abscess was stronger in those younger than 65 years than those older than 65 years (Table 2).

**Table 1:** Results from Cox proportional hazards regression model for association between diabetes status, glycemic control, body mass index, and risk of pyogenic liver abscess.

	No. of cases	Person-year	Incidence rate (/10,000 person-years)	Age- and sex-adjusted HR (95% CI)	Multivariable-adjusted HR (95% CI)
Glycemic control					
Non-diabetes	132	895,142.3	1.47	Ref	Ref
Diabetes	60	85,427.6	7.05	4.77 (3.52–6.47)	3.00 (2.18–4.13)
Good glycemic control (FPG≦130 mg/dl)	13	23,320.1	5.57	3.79 (2.14–6.70)	2.18 (1.22–3.90)
Poor glycemic control (FPG > 130 mg/dl)	47	62,107.6	7.57	5.14 (3.68–7.17)	3.34 (2.37–4.72)
Diabetes-related complications					
Non-diabetes	132	895,308.9	2.22	Ref	Ref
Diabetes					
DCSI = 0	8	12,776.5	6.26	4.26 (2.09–8.70)	3.42 (1.67–7.00)
DCSI = 1	12	14,915.9	8.05	5.47 (3.03–9.87)	4.22 (2.33–7.65)
DCSI > = 2	40	57,568.7	6.94	4.72 (3.31–6.72)	2.69 (1.85–3.89)
Body mass index (kg/m^2^)					
< 18.5	3	27,217.54	1.10	0.89 (0.28–2.81)	0.97 (0.31–3.09)
18.5–25.0	81	571,249.0	1.42	Ref	Ref
25.0–30.0	85	315,697.2	2.69	1.59 (1.18–2.17)	1.43 (1.05–1.95)
> 30.0	23	66,406.2	3.46	2.22 (1.40–3.53)	1.75 (1.09–2.81)

*HR* hazard ratio, *CI* confidence interval, *FPG* fasting plasma glucose, *DCSI* diabetes complications severity index.

*Adjusted for age, sex, tobacco smoking, alcohol use, betel nut use, education level, marital status, body mass index, biliary tract and gall bladder disease, cirrhosis, and GI/liver cancer. All variables were adjusted for as categorical variables (see Table 3 for details) except for age (as a continuous variable).

**Table 2:** Subgroup analyses on the association between diabetes status and risk of pyogenic liver abscess.

Covariates	Diabetes status	aHR* (95% CI)	*P* value**
Overall	No diabetes	Ref	
	Good glycemic control	2.35 (1.34–7.10)	
	Poor glycemic control	3.59 (2.54–5.06)	
Age			
< 65 years old	No diabetes	Ref	0.017
	Good glycemic control	3.08 (1.34–7.10)	
	Poor glycemic control	5.37 (3.52–8.21)	
≧ 65 years old	No diabetes	Ref	
	Good glycemic control	1.74 (0.78–3.84)	
	Poor glycemic control	2.00 (1.14–3.51)	
Sex			
Female	No diabetes	Ref	0.094
	Good glycemic control	2.56 (1.10–5.98)	
	Poor glycemic control	3.77 (2.31–6.15)	
Male	No diabetes	Ref	
	Good glycemic control	2.20 (1.00–4.82)	
	Poor glycemic control	3.44 (2.15–5.50)	
Biliary disease			
No biliary disease	No diabetes	Ref	0.106
	Good glycemic control	2.28 (1.25–4.17)	
	Poor glycemic control	3.29 (2.29–4.71)	
With biliary disease	No diabetes	Ref	
	Good glycemic control	4.89 (0.50–47.66)	
	Poor glycemic control	15.73 (3.75–66.00)	

*aHR* adjusted hazard ratio, *CI* confidence interval.

Good glycemic control: fasting plasma glucose ≦130 mg/dL.

Poor glycemic control: fasting plasma glucose > 130 mg /dL.

*Adjusted for age, sex, tobacco smoking, alcohol use, betel nut use, education level, marital status, body mass index, biliary tract and gall bladder disease, cirrhosis, and GI/liver cancer. All variables were adjusted for as categorical variables (see Table 3 for details) except for age (as a continuous variable).

**P value from likelihood ratio test.

**Table 3:** Baseline characteristics of the study population, stratified by diabetes status.

	Non-diabetes (n = 108,326)		Diabetes (n = 10,745)		Diabetes with good control* (n = 2974)		Diabetes with poor control* (n = 7771)		All (n = 119,071)	
Male	37,853	34.94%	4539	42.24%	1330	44.72%	3209	41.29%	42,392	35.60%
Age in year, median (IQR)	50 (16)		59 (14)		61 (15)		59 (14)		51 (16)	
Body mass index (kg/m^2^)										
< 18.5	3251	3.00%	100	0.93%	29	0.98%	71	0.91%	3351	2.81%
18.5 to 24.9	64,948	59.96%	4214	39.22%	1170	39.34%	3044	39.17%	69,162	58.08%
25 to 29.9	33,668	31.08%	4779	44.48%	1323	44.49%	3456	44.47%	38,447	32.29%
≥ 30	6459	5.96%	1652	15.37%	452	15.20%	1200	15.44%	8111	6.81%
Current smoker	15,869	14.65%	1747	16.26%	449	15.10%	1298	16.70%	17,616	14.79%
Current alcohol user	7520	6.94%	888	8.26%	211	7.09%	677	8.71%	8408	7.06%
Current betel nut user	2539	2.34%	291	2.71%	64	2.15%	227	2.92%	2830	2.38%
Marital status (married)	97,740	84.69%	8814	82.03%	2,411	81.07%	6403	82.40%	106,554	89.49%
Education (high school and above)	55,376	51.12%	3046	28.35%	869	29.22%	2177	28.01%	58,422	49.06%
Biliary tract and gallbladder disease	1283	1.18%	168	1.56%	56	1.88%	112	1.44%	1451	1.22%
Cirrhosis	430	0.40%	100	0.92%	42	1.41%	58	0.75%	530	0.45%
GI/liver cancer	534	0.49%	108	1.01%	46	1.55%	62	0.80%	642	0.54%

*IQR* interquartile range, *GI* gastrointestinal.

*Good glycemic control: fasting plasma glucose ≦130 mg/dl. Poor glycemic control: fasting plasma glucose > 130 mg/dl.

**Figure 3:**
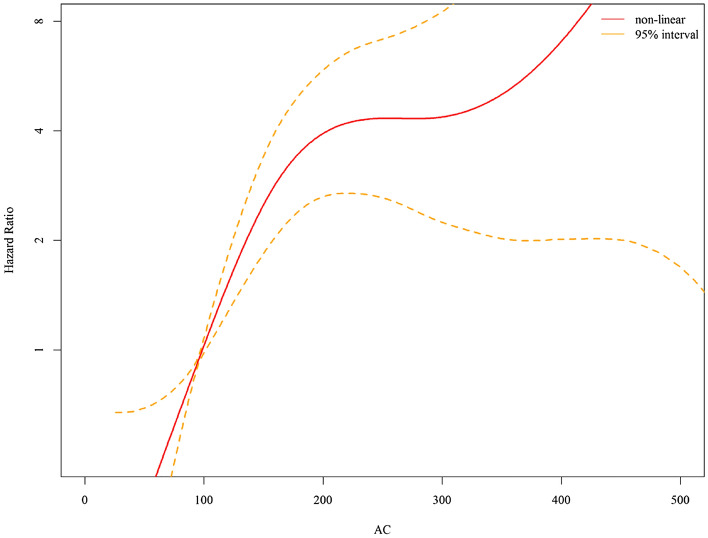
Dose–response curve between fasting plasma glucose and risk of pyogenic liver abscess. *Adjusted for age, sex, tobacco smoking, alcohol use, betel nut use, education level, marital status, body mass index, biliary tract and gall bladder disease, cirrhosis, and GI/liver cancer. All variables were adjusted for as categorical variables (see Table 3 for details) except for age (as a continuous variable).

In the linear dose–response analysis, the risk of liver abscess increased by 8.8% for every 10 mg/dl increase in FPG. Using spline regression, there was evidence for a non-linear relationship between FPG and the risk of liver abscess (p value for non-linearity: 0.0011). The risk of liver abscess increased sharply with increasing FPG for all values between 70 and 200 mg/dl, but the increase became less dramatic for FPG above 200 mg/dl (Fig. 3).

The overweight (adjusted HR: 1.43, 95% CI 1.05–1.95) and obese (adjusted HR: 1.75, 95% CI 1.09–2.81) populations were also found to have a higher risk of liver abscess when compared to people with normal weight (Table 1). The risk of liver abscess increased by 4.9% (95% CI 1.7–8.1%) for every one unit (kg/m^2^) increase in BMI. In the spline regression, there was no evidence of non-linear relationship between BMI and FPG (p value for non-linearity: 0.169).

In the population attributable fraction analysis, 15.4% and 16.0% of incident liver abscess in the study population could be attributed to diabetes and overweight/obesity respectively. In addition to diabetes and obesity, male sex (adjusted HR = 1.60, 95% CI 1.15–2.22), history of biliary tract and gallbladder disease (adjusted HR = 3.08, 95% CI 1.56–6.05), and liver cirrhosis (adjusted HR:3.08, 95% CI 1.13–8.41) were also significant risk factors for incident liver abscess in the multivariable Cox model.

## Discussion

Our large cohort study with long follow-up and adjustment for major confounding factors add to the evidence that improved glycemic control among diabetics may be associated with a reduced risk of pyogenic liver abscess. We also found that high BMI (overweight and obesity) was independently associated with an increased risk of pyogenic liver abscess, after adjusting for diabetes and other medical comorbidities including cirrhosis and biliary diseases. Thus, poor glycemic control and high BMI are linked to community-acquired liver abscess which is an emerging global disease punctuated by high mortality and morbidity.

There were few studies comparing the incidence of pyogenic liver abscess among diabetic populations compared to non-diabetic populations. In a Canadian study, the unadjusted risk ratio for pyogenic liver abscess was 11 when diabetics were compared to people without diabetes^27^. In 2004, an analysis of the Taiwan national health insurance database revealed that diabetic patients had a nine-fold increase in crude risk of pyogenic liver abscess when compared to non-diabetic patients^12^. In a population-based study in Denmark, patients with diabetes had a 3.6-fold increased risk of pyogenic liver abscess compared to population control subjects, after adjusting for age, sex, and medical comorbidities^28^. Using comprehensive social and demographic information and medical information, our study provided a robust estimate on the association between diabetes and risk of pyogenic liver abscess. The three-fold increase in risk of liver abscess after adjustment in our study was consistent with the adjusted estimate from Denmark.

A unique contribution of our study is investigating the association between glycemic control and the risk of pyogenic liver abscess. Compared to non-diabetics, the adjusted HR was 2.2 in diabetics with good glycemic control and 3.3 in those with poor glycemic control. To our knowledge this is the first study to report a potential reduction in risk of liver abscess if people have well-controlled diabetes. The link between glycemic control and the risk of common community-acquired infections has not been well explored in the literature^29^. Many studies on the relationship between glycemic control and infection risk were contradictory and/or underpowered, and did not adjust for confounders^4^. In a Taiwan single-center study of diabetic patients with confirmed pyogenic liver abscess, poor glycemic control was associated with increased frequency of cryptogenic liver abscess and metastatic infection^30^.

There appears to be a non-linear relationship between fasting plasma glucose (FPG) levels and the risk of liver abscess, with a sharp increase in risk between 70 and 200 mg/dl and after 300 mg/dl in our cohort. A previous study indicated that when glycemic control is very poor, defined by an HbA1c level greater than 11% (estimated average glucose of 269 mg/dL), the risk of severe infection is substantially elevated^31^. Further study may be needed to better understand the specific reasons for this non-linear relationship and the underlying mechanisms involved in the development of liver abscess in people with different level of high blood glucose. The risk of the liver abscess was stronger in those younger than 65 years than those older than 65 years was present in our study. This age attenuation can be seen in UK cohort in evaluation of overall long-term infection risk^31^. More comorbidity or unmeasured confounder in the elderly may explain this phenomenon.

In diabetic patients with good glycemic control, there was a “residual” hazard (adjusted HR: 2.18; 95% CI 1.22–3.90) for pyogenic liver abscess. We do not know if the residual hazard was a result of unmeasured confounding (e.g. by not controlling for socioeconomic status) or reflected a physiological mechanism. A previous bacteriologic study suggested that fecal colonization by hyper-virulent *K. pneumoniae* may be related to pyogenic liver abscess^32^. However, as we do not have the data to conduct this type of analysis, we are unable to determine whether diabetic patients in our study, regardless of their disease severity or control, had higher rates of fecal colonization from virulent strains or if any socioeconomic indicators impacted their the fecal microbiota. In addition to neutrophil dysfunctions related to hyperglycemia, other hyperglycemia-independent mechanism may explain the risk of infection or sepsis in diabetic patients^33^.

In contrast to glycemic control, diabetes-related complication were not associated with higher risk of pyogenic liver abscess. Conversely, diabetes duration and diabetic complications were related to urinary tract infection, but glycemic control did not have a linear relation with risk of urinary tract infection^34^. In a UK cohort, diabetes with chronic kidney disease or proteinuria was associated with a higher risk of pneumonia and sepsis^35^. Unlike pneumonia or urinary tract infection which may be related to diabetes complication or chronicity, our study suggested pyogenic liver abscess was more closely related to glycemic control. Further studies are needed to understand whether diabetes increases the risk of different sites of infections through different mechanisms^36^.

Previous studies showed that poor glycemic control impaired neutrophils phagocytosis of *K. pneumoniae* capsule serotypes K1 and K2^37^. A recent study from Taiwan showed that type 2 diabetes with poor glycemic control was a risk factor for disseminated Klebsiella infections^30^. Elevated glucose may enhance capsule-dependent evasion of the host immune system hence contributing to the development of disseminated infections^38^. On the other hand, a few animal studies indicated that host responses and immunity were related to increased susceptibility to *K. pneumoniae* infection in diabetic hosts. A study using mice model demonstrated that *K. pneumoniae* strains from pyogenic liver abscess exhibited higher virulence in diabetic than in nondiabetic mice and significantly decreased the blood cytokine TNF-alpha level^39^. Another study of hepatic responses to *K. pneumoniae* infection showed that morbidity and severity of hepatic tissue injury were enhanced in the diabetic mice, compared to the naïve mice^40^. The diabetic mice were more likely to develop *K. pneumoniae* liver infection. They were also more likely to have prolonged production of inflammatory mediator IL-1β, induction of ER stress, and increased hepatic apoptosis, which might contribute to *K. pneumoniae*-related hepatic damage. Therefore, interactions between bacterial capsule and host immune system, phagocytic functions, and host inflammation responses are likely involved in *K. pneumoniae*-related infection in a hyperglycemic host.

The linkage of obesity to pyogenic liver abscess in our cohort, after adjusting for confounding factors, is another novel finding. Most data of increase infection risk in obese populations examined nosocomial infection or surgical site infection^41^. In a cohort study of Danish women, obesity was associated with increased risk of community-acquired infections of the upper respiratory tract and skin^42^. There is no data on the risk of pyogenic liver abscess in obese population. In one drug-induced obese mice model and one leptin-deficient obese mice model, increased susceptibility to *Klebsiella pneumoniae* infections was found^43^^,^^44^. The possible mechanism of obesity linked to infection included altered cytokine synthesis, reduced antigen response, and diminished function of natural killer cells, dendritic cells, and macrophages^41^. Further studies are needed to investigate the association between obesity and risk of infection of different sites^45^.

One major strength of the present study is that we were able to adjust for several demographic and lifestyle risk factors as potential confounders in order to improve the causal interpretation of our findings (see the causal diagram in the Appendix Figure 1 for the assumed relationship among variables considered in our analysis). For example, smoking can increase the risk of gall stone, diverticular disease, colon cancer and inflammatory bowel disease, which may increase the risk of pyogenic liver abscess^46,47^, The risk of alcoholism related to liver abscess has been reported^48^. There were reports of chewing betel-nut (Areca catechu) increasing the risk of liver cirrhosis and liver cancer, which may then increase the risk of liver abscess^49^. And poor economic status was associated with increasing risk of pyogenic liver abscess in an India study^50^.

One limitation of the present study is the use of a single measurement of FPG at baseline as the indicator of glycemic control. Although previous studies showed a strong correlation between FPG and HbA1c, we did not have information on long-term glycemic control throughout the entire follow up period^51,52^. Nonetheless, we still observed a strong association between glycemic control measured at baseline and the risk of liver abscess. In addition, although we managed to adjust for major and known risk factors for pyogenic liver abscess, there is always a possibility of unmeasured confounding in an observational study. Lastly, competing risk could have occurred as 3.4% of the study participants died during the study period. The main results from the study were adjusted HRs from Cox regression modelling, which required the independent (i.e. non-informative) censoring assumption, i.e. that censored patients (due to mortality or lost to follow up) are “representative” of those under observation at the same time, conditioning on the covariates considered in the analysis^53^. Given the extensive data on the baseline covariate information, we believe this assumption would likely hold in our analysis. Nonetheless, the interpretation of Kaplan–Meier curve should be cautious, as it represents the hypothetical probability of disease-free survival in the absence of death.

In conclusion, poor glycemic control and higher BMI were associated with higher risk of pyogenic liver abscess in this large cohort study of the Taiwanese population. Improved glycemic control and weight reduction may therefore help to reduce the risk of the emerging infectious disease, pyogenic liver abscess.

## Supplementary Information

Supplementary Figure 1.

## Data Availability

The dataset of the current study is available from the corresponding author on reasonable request.
