# Supplementary material for: Diabetes and obesity and risk of pyogenic liver abscess
Source: Sci Rep. 2023 May 16;13:7922. doi: 10.1038/s41598-023-34889-z (PMC10188555; doi:10.1038/s41598-023-34889-z)
Supplement: Supplementary file 1 — Supplementary Figure 1. [file 41598_2023_34889_MOESM1_ESM.pdf]

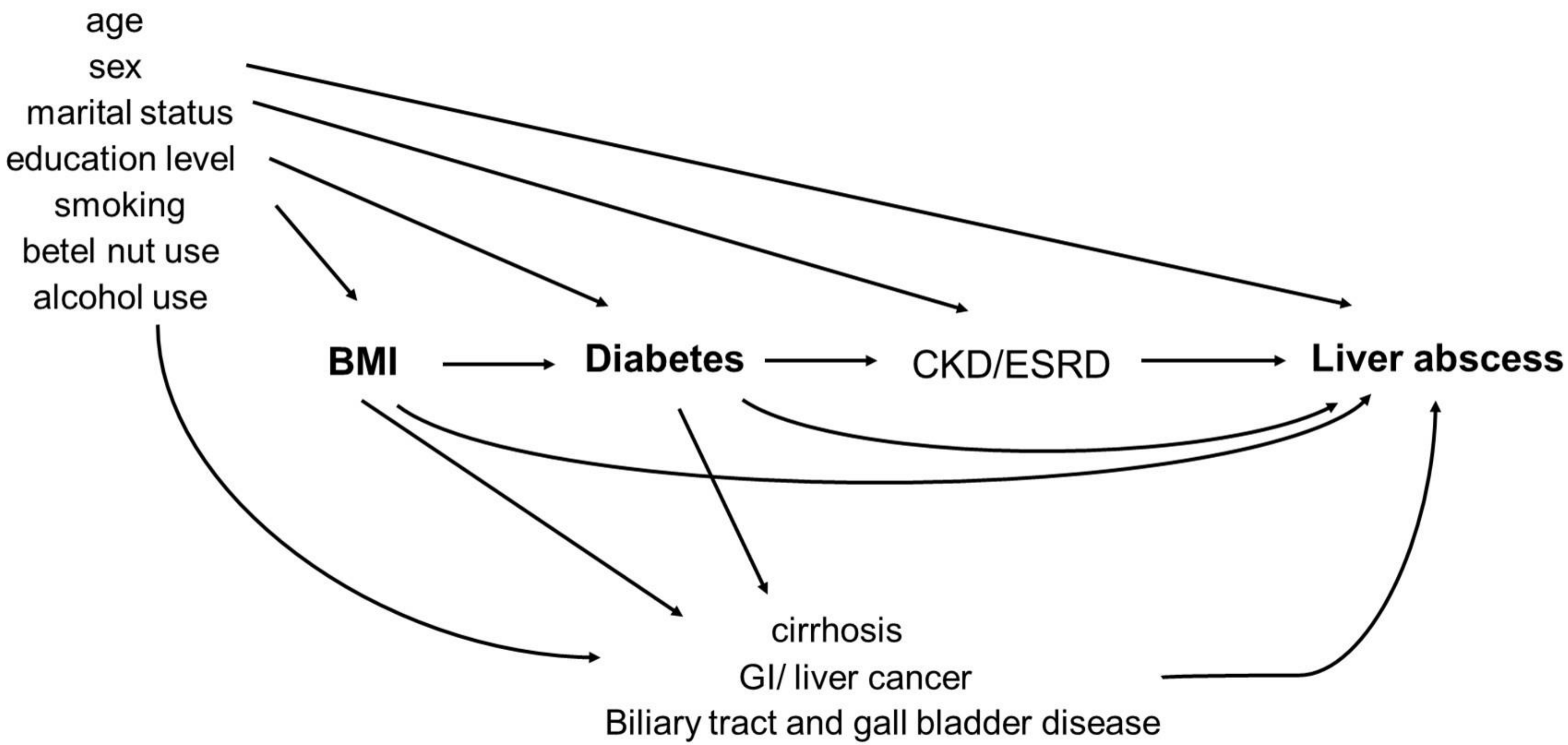

Appendix figure: Causal diagrams corresponding to the assumed relationships among variables considered in the study.
